# Stress and brain immunity: Microglial homeostasis through hypothalamus-pituitary-adrenal gland axis and sympathetic nervous system

**DOI:** 10.1016/j.bbih.2020.100111

**Published:** 2020-07-23

**Authors:** Shuei Sugama, Yoshihiko Kakinuma

**Affiliations:** Department of Physiology, Nippon Medical School, 1-1-5 Sendagi Bunkyo-ku, Tokyo, 113-8602, Japan

**Keywords:** Microglia, Stress, HPA axis, Smpathetic nervous system, Noradrenaline, Glucocorticoids

## Abstract

Stress has been well documented to bring about various clinical disorders, ranging from neurodegeneration, such as Parkinson’s (PD) and Alzheimer’s diseases (AD), to metabolic disorders including diabetes mellitus. Importantly, microglia, immunocompetent cells in the brain, have been shown to be involved in these clinical disorders. In the recent studies aiming to clarify the microglial responses, microglia are found to be quite responsive to stressful events, such as acute, subchronic, chronic stress, and social defeat stress. However, the mechanisms of these stress response on microglial activation have been not fully understood. In response to stress exposure, both the hypothalamic-pituitary-adrenal (HPA) axis and the sympathetic nervous system (SNS) are simultaneously activated, with the former inducing glucocorticoids (GCs) and the latter noradrenaline (NA), respectively. However, the effects of these stress-induced GCs and NA have not been consistent. The GCs, conventionally known to act on microglia as immunosuppressant, is also reported to act on it as stimulator. Similarly, the NA has been reported to act on microglia as stimulator or inhibitor depending on environmental conditions. Since any kinds of stress upregulate the HPA axis and SNS, with the levels of upregulation variable depending on the stress type, it is plausible that microglia is closely regulated by these two stress pathways. In this review, we discuss the microglial responses induced by various stresses as well as the possible mechanism by which stress induces microglial activation.

## Highlights

• The present study introduces the mechanism by which microglial activation occurs following acute stress.
• The present study suggests that microglial activation may be regulated through the HPA axis and sympathetic nervous system.
• The present study suggests that microglia may be inhibited by glucocorticoids, while activated by noradrenaline under physiological conditions.
• The present study suggests the hypothesis that the HPA axis may interact with sympathetic nervous system to maintain microglial homeostasis.

## 1 Introduction

Stress has been demonstrated to exert crucial roles in the susceptibility or the progression of several clinical disorders. Stress is conventionally well known to include a wide variety of physiological responses including the activation HPA axis and SNS. Upon exposure to stress, GCs are secreted from the adrenal cortex into the circulation, further into the brain through the blood-brain barrier (BBB). Consistent with the HPA axis activation, the SNS is activated to release neurotransmitters, such as NA, adrenaline (A), and acetylcholine (ACh) (Kventnansky et al., 1977, Kvetnanský et al., 1995; Turnbull and Rivier, 1999; Sabban and Kventnansky, 2001; McEwen et al., 2015; Sapolsky, 2015). Thus, it is highly likely that the stress response may regulate the brain immunity through the hormone and neurotransmitter.

Microglia are immunocompetent cells in the brain that contribute to a wide variety of roles, such as monitoring or nurturing neuronal activity, structural remodeling of neurons, surveillance of the neuronal milieu, neuroinflammation, phagocytosis, and gliosis. While they usually exhibit a ramified morphology, with long dendrites and small cell somas under control conditions, they are activated by various stimuli, including axotomy, inflammation, and neuronal damage (Kreutzberg, 1996; Graeber, 2010). The microglia can be morphologically categorized into several types: ramified, hyper-ramified primed, reactive, and phagocytic, with phagocytic cells further differentiating into transitional (T-stage), motile (M-stage), and locomotor (L-stage) (Stence et al., 2001; Walker et al., 2013). In addition to the morphological changes (Perry et al., 1987; Stoll et al., 1989; George and Griffin, 1994), they occasionally release cytotoxic factors, such as nitric oxide (NO), cytokines, chemokines, and reactive oxygen species (ROS) (Block and Hong, 2005; Gao et al., 2008; Lenz et al., 2013).

From earlier time, it has been shown that microglia play crucial roles in various neurodegenerative diseases, such as AD and PD. For instance, in animal model of AD, microglial activation has been demonstrated surrounding the senile plaques (Rozemuller et al., 1989). Microglia around the senile plaques are also demonstrated to release pro-inflammatory cytokines, such as interleukin (IL)-1β, Il-6, and TNFα (Dickson et al., 1993; Griffin et al., 1995; Kaplin et al., 2009). In animal model of PD, robust activated microglia have been observed in the substantia nigra (SN) surrounding dopaminergic neurons (Czlonkowska et al., 1996; Kohutnicka et al., 1998; Kurkowska-Jastrzebska et al., 1999; Wu et al., 2003; Sugama et al., 2003; Hu et al., 2008). Recently, it is also suggested that microglia are involved in psychiatric disorders, such as depression (Steiner et al., 2008; Torres-Platas et al., 2014; Yirmia et al., 2015), bipolar disorder (Haarman et al., 2014), insomnia (Huang et al., 2014), and autism (Tetreault et al., 2012). Thus, microglial activation contributes to various neurological disorders. On the other hand, however, microglia release neurotrophic factors, exerting neuroprotective roles. This is a reason why microglia are called “double-edged sword.” Thus, maintaining the microglia in “quiescent” condition is critical in the normal brain activity.

Intriguingly, microglia are found to be quite responsive to various stressful events, such as acute stress (Sugama et al., 2007; Frank et al., 2007; Yoshii et al., 2017), repeated stress (Nair et al., 2006; Tynan et al., 2010; Sugama et al., 2016; Pietrogrande et al., 2018), and social defeat (Wohleb et al., 2011; Lehmann et al., 2016; Stein et al., 2017). The stressful events have been shown to significantly upregulate the HPA axis and SNS, with their various upregulation levels depending on each stress type. Although each individual differs in the level of responses depending on personality factor, mood, age, gender, or even conditions (Kunz-Ebrecht et al., 2003), it is plausible that microglia might receive the “common” signals generated by those stressful events.

However, the mechanism governing stress-induced microglial activation is not fully understood. The main goal of this review is to discuss this point, the mechanism how stress could modulate the brain immunity focusing on microglial cells.

## 2 The effect of stress on microglial activation

### 2.1 Effect of acute stress on microglial cells

Microglia have been well documented in relation to neuroinflammation. It is in the past 15 years that the relationship between stress and microglial responses becomes clearer. The first two reports were published in 2007 from different laboratories which specifically focused on the effect of acute stress on microglial cells. At first, Frank et al. exposed 3-month-old male Sprague Dawley (SD) rats to inescapable tail shock, with 1.6 ​mA, 5 ​s each, 100 times. They found the increase of MHC II-positive cells in the dentate gyrus and CA3 region in the hippocampus (HC). Eventually, these MHC II-positive cells were found to be Iba1-positive microglia (Frank et al., 2007). Intriguingly, they could not see any changes in GFAP-positive astrocytes, nor CD11b-immunoreactive (ir) microglial cells. On the other hand, Sugama et al. employed severe stresses, restraint stress combined with water immersion stress, to 3-month-male Wistar rats. The authors found that morphological hypertrophic changes of CD11b-ir microglia occurred within 1 ​h to the HT, thalamus (TM), HC, and other brain regions (Sugama et al., 2007). The finding of microglia being activated within 30 ​min of acute stress indicates the involvement of fast signals, such as those conveyed by neurotransmitters. In addition, morphological microglial activation was neither accompanied with elevation of IL-1β, IL-6, or iNOS mRNA nor with ED1 (phagocytic marker) and OX-6 (MHC II marker) (Sugama et al., 2007).

Thus, acute stress-induced microglial activation was demonstrated by MHC II expression (Frank et al., 2007) and morphological hypertrophy (Sugama et al., 2007). In fact, microglial activation is generally characterized with morphological changes and functional activations. Once morphologically activated, microglia release neurotoxic factors, such as NO, cytokines, chemokines, and ROS. Therefore, it is not clear at this stage whether stress-induced microglia are anti-inflammatory or pro-inflammatory or when the stress-induced microglia turn to feature inflammatory properties. Possible explanation could be that, at the early phase of acute stress, microglia may not show any inflammatory markers (Sugama et al., 2007). However, the microglia may become inflammatory depending on the environmental conditions, such as less sufficient immunosuppressive hormones like GCs, the continuation of severe stress exposures, or the existence of neurodegenerative changes.

## 3 Effect of subchronic and/or chronic stress on microglial cells

Stress-induced microglial activation has also been observed in subchronic and/or chronic stress models. In this review, we classified the stress within 7 days as subchronic and the one longer than 7 days as chronic. It was Nair’s report which demonstrated that subchronic stress induces microglial proliferation (Nair et al., 2006). They exposed C57BL/6 mice to 15-h restraint stress for the 6 straight days. In the experiment, they found that the number of microglia significantly increased on the 4th day of the stress. In addition, they found corticosterone levels were correlated with the increase of microglia. Furthermore, they demonstrated the increase of microglial cells following corticosterone administration via ingestions of corticosterone dissolve in water (400 ​μg/mL, 1400 ​μg/kg). They further demonstrated that MK-801, an NMDA receptor antagonist, significantly inhibited microglial proliferation, leading to a conclusion that increased corticosterone levels stimulate NMDA receptor, resulting in microglial proliferation. Similar results were also reported with subchronic stress. It was also demonstrated by a different group that repeated restraint stress, 2 ​h a day for 4 straight days, significantly increased OX-42-ir microglia in the CA1 and striatum in ICR mouse (Kwon et al., 2008). In the study, IL-1β was detected not in microglia, but in neuronal cells (Kwon et al., 2008).

As for chronic stress, it was Tynan’s report which systemically confirmed the stress-induced microglial activation (Tynan et al., 2010). They exposed SD rats to chronic stress, 30 ​min unpredicted stress in one-shot stress, twice a day for 2 weeks. They studied 15 brain regions, including the cerebral cortex (CCx), nucleus accumbens (NAc), striatum (STR), HT, amygdala (Amy), HC, periaqueductal central grey (PAG), and ventral tegmental area (VTA). They found that microglial number was significantly increased in the CCx, NAc, STR, and PAG (Tynan et al., 2010). In those regions, morphological alterations were also observed, however, with MHC II undetected. In addition, they also attributed the increase of microglial cells to the increased levels of CD11b immunoreactivity levels since Ki67, a marker for cell proliferation, was shown to be negative. Furthermore, Hinwood et al. exposed SD rats to chronic stress, restraint stress for 6 ​h a day, for 21 day. They found morphological microglial activation in the prefrontal cortex (PFC). Importantly, the activated microglia were not positive to MHC II, CD 68, caspase-3, or TUNEL (Hinwood et al., 2012). With a hypothesis that the microglial activation in the PFC may be involved in spatial working memory, they tested the ability of a microglial activation inhibitor (minocycline). They found that minocycline reduced the impact of stress on working memory disturbance as well as microglial activation, suggesting that microglia may play a role in mediated PFC-regulated behavior (Hinwood et al., 2012).

Recently, other types of chronic stress have been tested. Wang et al. exposed SD rats to the chronic stress which comprises of nine different stressors: water deprivation, food deprivation, light/dark cycle reversal, hot environment (40 ​°C, 5 ​min), swimming in cold water (4 ​°C, 5 ​min), cage shake (15 ​min), restraint (2 ​h), radio noise (12 ​h). These stressors were loaded once a day for 12 weeks. They detected microglial activation in the HC by a PET study. They found that chronic minocycline treatment alleviated the depressive-like behavior induced by chronic stress and significantly inhibited microglial activation (Wang et al., 2018). In addition, Lio et al. exposed C57BL/6 mice to chronic stress, 2 ​h restraint stress per day, for one week. They detected microglial activation in the Amy. They found that chronic minocycline treatment inhibited microglial activation and reduces the anxiety-like behaviors (Liu et al., 2018).

## 4 Microglial modulation following stress through GCs

**Fig. 1:**
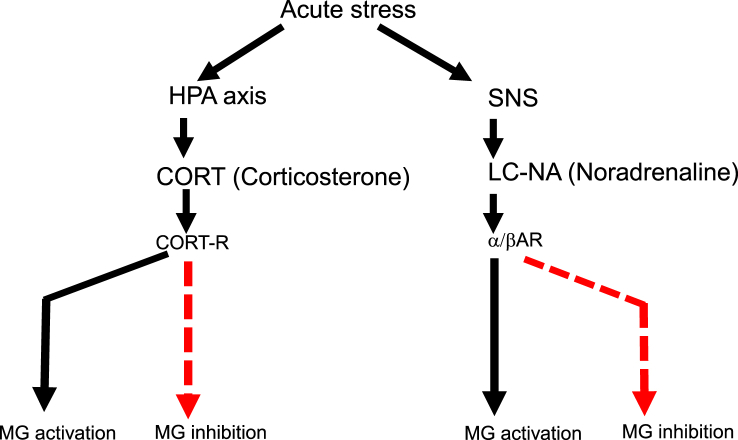
Schematic depict of microglial activation through the SNS and HPA axis. Microglial activation is induced by NA and GCs, respectively. By contrast, microglial inhibition is induced by NA and GCs as well. Solid line (black) indicates “stimulation,” while dotted line (red) indicates “inhibition.”. (For interpretation of the references to color in this figure legend, the reader is referred to the Web version of this article.)

From the early studies regarding stress vs. microglia, GCs are the most-investigated candidate to induce stress responses especially through HPA axis (Fig. 1). Therefore, it is critical to clarify the possible effects of this hormone on brain immunity.

GCs are the steroid hormones which predominantly affect the metabolism of carbohydrate and protein, in contrast to the mineralocorticoids which largely influence sodium absorption and potassium excretion. They are exclusively secreted from the adrenal cortex. Among innumerable steroids isolated from the adrenal gland, the hormones secreted in physiologically significant amounts are cortisol and corticosterone as GCs, while aldosterone as mineralocorticoids. Importantly, the ratio of corticosterone and cortisol varies depending on species. For instance, in mice and rats, corticosterone exclusively functions, while in humans cortisol exerts as GCs. Therefore, in most cases, GCs correspond to corticosterone in rodent, while cortisol in humans.

In mammals, 90% GCs are bound to corticosteroid binding globulin (CBG), with the rest of the 10% being free. Importantly, only free GCs cross the blood–brain barrier (BBB), physiologically functioning in brain immunity. The protein-bound GCs are considered to work as circulating reservoir of hormone. Since the CBG is synthesized in the liver and increased by estrogen hormone, the levels of free GCs can be modulated by impaired protein synthesis such as in liver cirrhosis, massive protein loss, nephrotic syndrome, pregnancy, or gender difference.

Tanaka et al. reported the microglia possess GCs receptor (GR) (Tanaka et al., 1997). There being a number of reports regarding the influence of GCs on microglial activation, they are mainly grouped into two major functions: pro-inflammatory and anti-inflammatory (Fig. 1).

For instance, Dinkel et al. investigated male SD rats focusing on kainic acid (KA)-induced inflammatory reactions in the brain (Dinkel et al., 2003). Three different groups, such as intact control, adrenalectomized (ADX)/GCs-supplemented, and GCs-treated, were injected with KA into the hippocampal CA3 region. The animals had GCs levels at 28 ​μg/dL, the baseline of which being 1–10 ​μg/dL. Intriguingly, GCs treatment increased the numbers of inflammatory cells including granulocytes, monocytes/macrophages, and microglia in the lesion site. Importantly, the expression of mRNA and protein levels of IL-1α, IL-1β, and TNFα were significantly increased by GCs (Dinkel et al., 2003). Consistent with this result, McPherson et al. investigated the effects of corticosterone on the KA-induced inflammatory responses in cultured hippocampal neurons. They found that corticosterone (1 ​μM) significantly increased the production of IL-1β, TNFα in the inflammatory reactions (MacPherson et al., 2005). The concentration of corticosterone 1 ​μM is considered to be the highest limit of steroid hormone that can be used in primary cultures and equated with maximal stress concentration of corticosterone in vivo (Sapolsky et al., 1995). In addition, from a neurodegenerative point of view, De Pablos et al. studied the involvement of GCs in the stress-induced dopaminergic neurodegeneration using Wistar rats (de Pablos et al., 2014). They exposed rats to chronic variate stress treatment for 9 days which comprises of forced swimming, restraint, water deprivation, restraint at 4 ​°C, isolation, and food deprivation. At first, they found that the chronic stress significantly exacerbated dopaminergic neurodegeneration which was induced by LPS (2 ​μg) injection into the SN. Importantly, the dopaminergic neurodegeneration was significantly inhibited by RU486 (20 ​mg/kg), a potent inhibitor of GR, through the suppression of microglial activation as well as the reduction of pro-inflammatory markers, such as TNFα, IL-1β, IL-6, and iNOS (de Pablos et al., 2014). Thus, these results indicated the pro-inflammatory roles of GCs through GR in the SN.

On the other hand, a number of studies demonstrated anti-inflammatory roles of GCs in the brain. For instance, Nadeau and Rivest investigated the effect of RU486 (50 ​mg/kg/200 ​μl), GR inhibitor mifepristone, on LPS (5 μg/2 ​μl)-induced brain inflammation. They found that the RU486 administration increased the inflammatory reactions, such as TNFα, IL-1β, and caspase-8, indicating the anti-inflammatory roles of GCs (Nadeau and Rivest, 2002). In addition, Nadeau and Rivest further demonstrated that the increased levels of circulating GCs, induced by pretreatment with LPS injection, significantly inhibited TNFα, IκBα, and MCP-1 mRNA expression in the LPS-induced brain injury (Nadeau and Rivest, 2003). The inhibition of GCs synthesis via injection of metyrapone (50 ​mg/kg s. c.) increased IL-1 mRNA in the HT which is provoked by foot-shock stress (Blandino et al., 2006). In animal model of PD, GCs have been tested for microglial activation and neurodegeneration. Kurkowska-Jastrzebska et al. investigated the effects of dexamethasone (DXM) on MPTP-induced dopaminergic neurodegeneration. They found that DXM significantly inhibited the infiltration of lymphocytic cells, such as CD3, CD4, and CD8, into the SN, resulting in the neuroprotection (Kurkowska-Jastrzebska et al., 1999, 2004). Similarly, anti-inflammatory effects of GCs were shown on the MPTP-induced dopaminergic neurodegeneration using GR-deficient mice (Molale et al., 2004), ADX mice (Sugama et al., 2009), and GR mutant mice (GR^LysMCre^) (Ros-Bernal et al., 2011). Moreover, with cultured microglial cells, DXM is shown to suppress LPS-induced NF-kB activation in the brain (Glezer et al., 2003). Consistently, in primary microglial cells, cortisol represses LPS induction of NO, iNOS, and TNFα (Drew and Chavis, 2000).

To address the role of microglial cells in acute stress, we studied the response following acute stress on three different conditions: sham (SHM), ADX, and ADX plus exogenous corticosterone administration (ADXC) (Sugama et al., 2013). We demonstrated that OX-42 (CD11b)-ir microglia were robustly enhanced in ADX rats, while corticosterone treatment significantly reduced the effect of adrenalectomy, suggesting that GCs may serve an important endogenous suppressive signal limiting neuroinflammation that otherwise might occur during stress (Sugama et al., 2013). This result is also consistent with the finding that increased pro-inflammatory cytokines, microglial activation, and oxidative stress occur in the HC following the bilateral ADX operation (Nguyen et al., 1998; Hamadi et al., 2016). These results suggest that GCs may play anti-inflammatory roles in microglial activation (Baker et al., 2001; Kunz-Ebrecht et al., 2003).

## 5 Opposite effects of GCs on microglia

With regard to the controversial effects of GCs, it is absolutely important to specify the possible mechanisms. At this stage, there are several possible explanations.

First, GCs concentration may matter (Walker et al., 2013). MacPherson demonstrated that low-to midrange GCs concentrations (0.2–0.6 ​μM) decreased expression of IL-1β and TNFα, while the higher GCs doses (1 ​μM) potentiated the expression of these pro-inflammatory cytokines. This result suggests that low to mid-dose of GCs is anti-inflammatory, while high dose is pro-inflammatory (MacPherson et al., 2005; Munhoz et al., 2010; Sapolsky, 2015).

Second, the timing of GCs exposure may matter (Walker et al., 2013). Frank et al. demonstrated that GCs potentiated the brain pro-inflammatory responses to an LPS challenge if GCs are administered prior, 2–24 ​h, to challenge. In contrast, when GCs are administered after the LPS challenge, GCs suppressed the pro-inflammatory responses (Frank et al., 2010). This result is explained by the possibility that GCs sensitize microglial cells to become more sensitive to the coming challenge, be it LPS or other neurotoxins. In particular, CD200-CD200R1 signaling contributes to the checkpoint mechanisms which serve to inhibit the activity of microglial cells. Frank et al. clearly demonstrated that exposures to stress significantly decreased CD200R, eventually leading to the microglial activation (Frank et al., 2018). Indeed, in Dinkel’s study showing pro-inflammatory effects of GCs, GCs were administered prior to KA challenge (Dinkel et al., 2003). In addition, in Smyth’s study showing pro-inflammatory reactions by GCs as well, corticosterone was added to cultured macrophages 12 ​h before LPS challenge (Smyth et al., 2004).

Third, the GCs response may be impaired due to the downregulation of GR or other reasons (Kunz-Ebrecht et al., 2003). A previous study, comparing the plasma levels of IL-6 and IL-1ra between cortisol nonresponder and responder groups, demonstrated greater levels of IL-6 and IL-1ra in a cortisol nonresponder group (Kunz-Ebrecht et al., 2003). Individuals with impaired cortisol response are considered as cortisol nonresponder. Thus, it is possible that unresponsiveness caused by the downregulation of GR might account for differential effects of GCs on microglia.

## 6 Microglial modulation through LC-NA system

NA is the best documented neurotransmitter in stress experiments. In fact, NA is reported to increase in the brain in response to various types of stresses, such as immobilization, foot shock, or tail pinch (Tanaka et al., 1991; Page et al., 1997).

NA is synthesized from dopamine (DA) by DA-β-hydroxylase, DBH. DA is originally synthesized from tyrosine by the rate-limiting enzyme tyrosine hydroxylase (TH). Therefore, among the TH-containing neurons which are potentially synthesizing either DA or NA, only the DBH-containing neurons are considered to specifically yield NA. The effects of NA are mediated by three types of G-protein coupled receptors, α1, α2, and β, with β further divided into β1, β2, and β3. While the α1 and β receptors are present at postsynaptic site, α2 receptors are at both pre- and postsynaptic sites (Benarroch, 2017).

In terms of a neuroanatomical point, the LC, located in the upper dorsolateral pontine tegmentum, is the largest nucleus which synthesizes and projects NA throughout the brain. Extensive branched axons provide the main source of NA throughout the brain including neocortex, Amy, cerebellum, and spinal cord (Lindvall and Bjorklund, 1974; Mello et al., 1998). Therefore, NA is much more ubiquitously distributed in the brain than any other catecholamines, such as A, DA, serotonin (5HT), or ACh.

Microglia, ubiquitously distributed throughout the brain, possess α- and β-adrenergic receptors (ARs) (Mori et al., 2002). The AR receives signal from a ligand, NA or A. In contrast to the periphery where A outnumbers NA, in the brain, the amount of NA is estimated to be more than several times as much as that of A. The A is converted from NA by phenylethanolamine N-methyltransferase (PNMT), the enzyme found in appreciable quantities only in the brain and the adrenal medulla. Since PNMT is detected in the HT (Trocewicz et al., 1982; Algeri et al., 1988), a certain amount of A may function as ligand in the HT.

As to the role of NA for neuroimmunomodulation, there have been a number of reports suggesting either pro-inflammatory or anti-inflammatory roles of NA (Fig. 1). For instance, administration of β-AR agonist, isoproterenol, significantly increased IL-1β in the brain (Yabuuchi et al., 1997; Johnson et al., 2005) and cultured microglia (Tomozawa et al., 1995). Besides, microglial activation induced by repeated social defeat is completely blocked by propranolol (10 ​mg/kg), an antagonist of β1-AR and β2-AR (Wohleb et al., 2011). In addition, the induction of IL-1β in the HT by foot-shock stress is blocked by propranolol (20 ​mg/kg) with SD rats (Blandino et al., 2006, 2009), which also inhibits pro-inflammatory cytokine production in microglial cells isolated from rats (Wang et al., 2010). In our recent study, we found that β-blocker treatment, propranolol (10 ​mg/kg), inhibited microglial activation in terms of morphology and cell count through the whole brain; however, α-blockers, prazosin or yohimbine, did not show such effect. Furthermore, unlike WT mice, double knockout mice which lack β1-AR and β2-AR exhibited substantial inhibition of stress-induced microglial activation in the brain (Sugama et al., 2019). Moreover, ablation of LC-NA system is shown to prevent stress-induced elevation of IL-1β in the HC, a region highly innervated by the LC (Johnson et al., 2005). These results suggest pro-inflammatory roles of NA via β1-AR and β2-AR.

On the contrary, it is also true that several studies reported the anti-inflammatory effects of NA, which is an opposite finding to our current study. For instance, in cultured microglia, NA and isoproterenol, β-AR agonist, inhibit proinflammatory markers, such as IL-1β, IL-6, and iNOS mRNA following LPS treatment (1 ​μg/mL) through the inhibition of NFkB translocation (Ishii et al., 2015). In addition, in the HC of aged rats in which LC-NE were beforehand lesioned by DSP4 (25 ​mg/kg), a neurotoxin capable of crossing the BBB and toxic to NE neurons, LPS treatment (0.75 ​mg/kg) induced the aggravation of inflammation (Bharani et al., 2017). Also, in the SN of mice in which LC-NE were lesioned by DSP4 (20 ​mg/kg, 4 times), MPTP treatment induces more dopaminergic neurodegeneration as well as microglial activation than control mice (Yao et al., 2015). These results suggest that NE may protect neurons through inhibiting microglial cells.

## 7 Opposite effects of NA on microglia

Such differential effect of NA, anti- or pro-inflammatory, has been one of intriguing paradoxes (Fig. 1). However, one possible reason is attributable to the differential pathway which β-AR induces following NA binding.

Previous studies demonstrated that NA–β2-AR axis inhibits LPS-induced induction of cytokines, such as IL-1β, IL-6, and TNFα, through the inhibition of NFκB in human monocyte lineage, THP-1 ​cells (Farmer and Pugin, 2000; Severn et al., 1992). Isoproterenol, a specific β-AR agonist at 0.01 ​nM, suppressed TNF production in THP-1 ​cells (Severn et al., 1992). In addition, in animal experiments of Fischer 344 rats, propranolol (10 ​mg/kg) administration prevented the IL-1 and IL-6 from increasing following tail-shock stress in the HT and HC (Johnson et al., 2005). At intracellular signaling levels, binding of NA to β-AR increases cAMP levels which eventually inhibit the pro-inflammatory responses through protein kinase A (PKA) (Housay et al., 2005). Thus, the anti-inflammatory effects of NA are attributed to PKA-dependent pathway.

On the other hand, Qian et al. demonstrated that NA–β2-AR axis selectively induced dopaminergic neurotoxicity through microglial activation (Qian et al., 2009). They showed that β2-AR activation increased the production of ROS by NADPH oxidase (PHOX), the mechanism of which reveals that β2-AR induces microglia PHOX activation and neurotoxicity through an ERK-dependent/PKA-independent pathway (Qian et al., 2009). In addition, another report from the same laboratory demonstrated that β2-AR stimulation increased the production of pro-inflammatory mediators in both macrophages and microglial cells (Tan et al., 2007). Consistently, in our recent study, we found that propranolol (10 ​mg/kg), β-blocker, significantly inhibited pro-inflammatory cytokine production, such as IL-18 and IL-1β, as well as Iba1-ir microglia (Sugama et al., 2019). This result is consistent with the previous findings that β-AR agonist, isoproterenol, significantly increased IL-1β in the brain (Yabuuchi et al., 1997), as well as in cultured microglia (Tomozawa et al., 1995), that a β-blocker, propranolol (20 ​mg/kg), blocked the foot shock-induced production of IL-1β in the HT (Blandino et al., 2006), and that β-blocker (10 ​mg/kg) inhibited microglial activation which is induced by repeated social defeat stress (Wohleb et al., 2011). Although precise mechanism is not yet understood, it is conceivable that an alternative pathway, independent of PKA/NFκB mechanism, may be involved in pro-inflammatory effects of NA.

## 8 Microglial homeostasis through HPA axis and LC-NA system

**Fig. 2:**
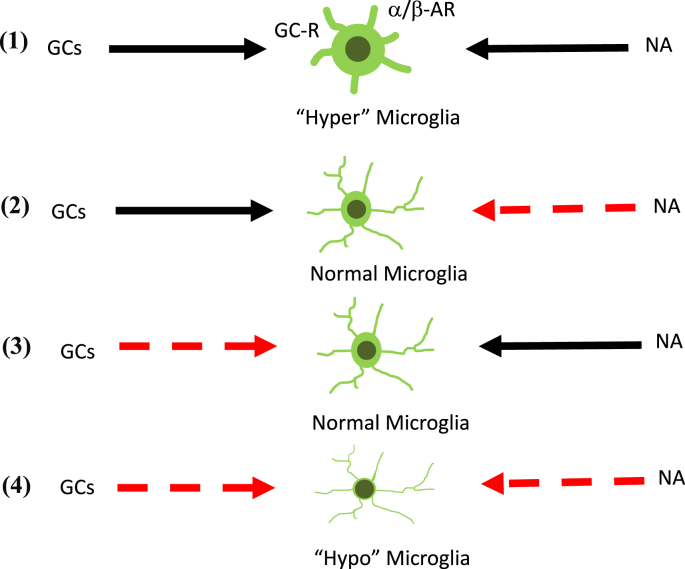
Schematic depict of microglial activation pattern through the SNS and HPA axis. Microglia show “hyper” (1) conditions as a result of simultaneous stimulation and “hypo” (4) condition as a result of simultaneous inhibition through the SNS and HPA axis, respectively. Microglia are controlled in the way which the SNS and HPA axis harmoniously constitute as shown in (2) and (3). Solid line (black) indicates “stimulation,” while dotted line (red) indicates “inhibition.”. (For interpretation of the references to color in this figure legend, the reader is referred to the Web version of this article.)

With regard to the effect of stress on microglial cells, there are limited possible scenarios. Regardless of the mechanisms by which GCs and NA affect microglial cells, there are at least four patterns as follows: 1) GCs as microglial stimulator, NA as a stimulator as well; 2) GCs as microglial stimulator, NA as an inhibitor; 3) GCs as microglial inhibitor, NA as a stimulator; 4) GCs as microglial inhibitor, NA as an inhibitor as well (Fig. 2).

First, in case of the setting (1), stress induces microglial activation, without any inhibitory mechanisms. Similarly, in case of setting (4), stress may inhibit microglia, without any stimulatory signals. In these two cases, it is likely that the stress exposure induces either a “hyper” or “hypo” microglial condition, both of them putting a glial condition to the extreme status resulting in pathological conditions (Fig. 2) (Stein et al., 2017). These two extreme statuses could alter the mood levels into depressive conditions (Yirmia et al., 2015). Obviously, these statuses do not support a homeostatic point of view. Thus, these two settings are less likely to account for the stress response under physiological conditions.

Next, when we consider the balance of microglial status, there remains two patterns as follows: 2) GCs as microglial stimulator, NA as an inhibitor and 3) GCs as microglial inhibitor, NA as a stimulator (Fig. 2). Theoretically only in those patterns, the microglia could be well balanced by the HPA axis and LC-NA system. Therefore, the crux appears to rely on the interaction between HPA axis and LC-NA system.

## 9 Interactions between HPA axis and LC-NA system

In fact, there have been a number of reports demonstrating the mutual interactions between the two stress systems (Page et al., 1997). Harfstrand et al. identified GR in the noradrenergic (A1, A2, A4, A5, A6, and A7), adrenergic (C1, C2, and C3), serotonergic (B1–B9), and dopaminergic (A8, A9, A10) neurons in the brain stem by using two-color immunocytochemistry (Harfstrand et al., 1986). In particular, vast numbers of GR-ir nuclei were shown in the LC classified as A6. Interestingly, the strongest GR immunoreactivity was detected on the dopaminergic cells classified A10, also called VTA, the nucleus highly responsive to stress. It clearly suggests that ascending noradrenergic neurons may receive the signal of GCs (Harfstrand et al., 1986; Sawchenko and Bohn, 1989).

On the other hand, although the LC, A6, does not deliver direct projections to the hypophysiotropic neurons of the PVN (Cunningham, 1988; Sawchenko and Swanson, 1981, 1982), it projects to various stress-related regions, such as PFC, Amy, and HC, which are interconnected with the parvocellular and magnocellular PVN (Herman and Cullinan, 1997).

**Fig. 3:**
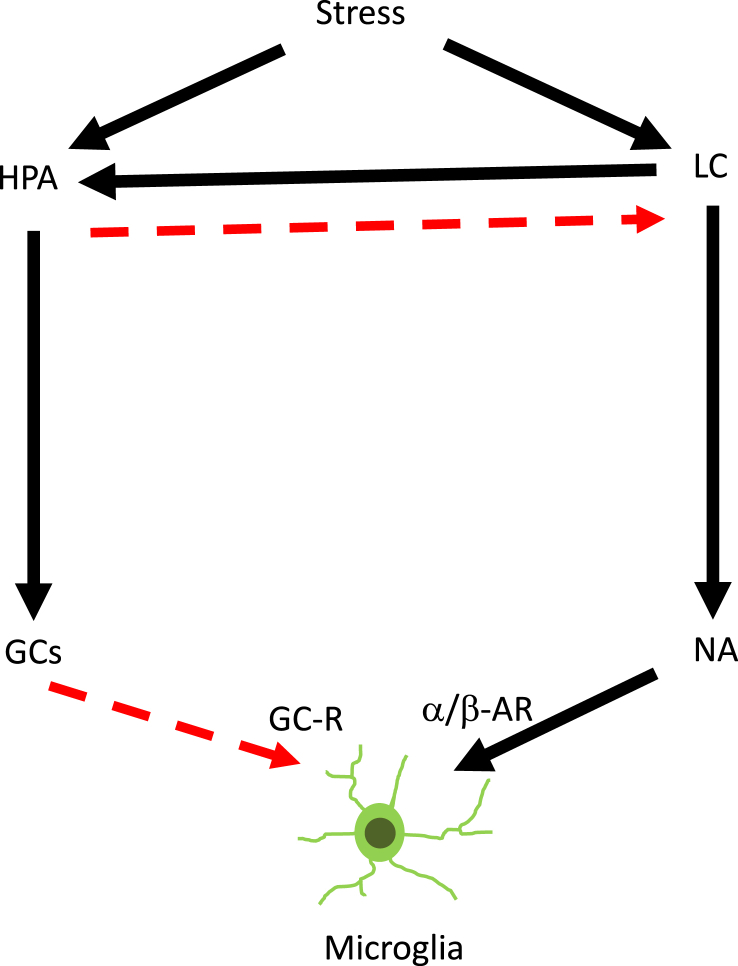
Schematic depict of microglial regulation through the SNS and HPA axis. Microglial status is regulated during stress exposure through the SNS and HPA axis, two stress responsive systems interacting each other. Solid line (black) indicates “stimulation,” while dotted line (red) indicates “inhibition.”. (For interpretation of the references to color in this figure legend, the reader is referred to the Web version of this article.)

From the functional point of view, Ziegler et al. investigated the effect of LC-NA systems on the levels of plasma corticosterone using SD rats. In the rats whose bilateral LC was injured by 6-hydroxydopamine (6-OHDA) injection into the nucleus, the levels of plasma corticosterone were significantly decreased as compared with control rats. They clearly demonstrated that the lesion of LC-NA system eventually lead to the suppression of HPA axis, indicating that LC-NA system under normal conditions may serve signals to activate HPA axis (Ziegler et al., 1999). By contrast, Pacak et al. investigated the effect of HPA axis on the levels of NA in the brain by using SD rats. In the rats whose bilateral adrenal glands are removed by ADX procedure, the levels of NA were significantly upregulated in microdialysis study. They successfully demonstrated that the ADX lead to the increase of NA levels, indicating that HPA axis may under control conditions inhibit LC-NA system (Pacak et al., 1993; Kventansky et al., 1995; Makino et al., 2002). Thus, these results suggest that HPA axis inhibits the LC-NA system, while LC-NA system activates the HPA axis, maintaining the homeostasis in response to stress exposures (Fig. 3).

However, when it comes to the effects on microglial cells, only a few studies reported the effects of these two systems on the microglial activation. Based on previous studies including ours, microglial activation was enhanced in ADX (Sugama et al., 2013; Hamadi et al., 2016), while it was blocked by the administration of β-blocker and gene knockout of β1-and β2-AR (Blandino et al., 2006; Wohleb et al., 2011; Sugama et al., 2019). Recently, Barnard et al. investigated the effects of NA and GCs on microglial activation using β-blocker, propranolol (10 ​mg/kg), and GCs synthesis inhibitor, metyrapone (100 ​mg/kg). They found that, in male rats, inhibiting the synthesis of GCs by metyrapone resulted in widespread increases of IL-1β mRNA throughout the brain, and that propranolol (10 ​mg/kg) administration inhibited IL-1β mRNA in the HC, Amy, and PFC (Barnard et al., 2019). Thus, these results suggest that NA may stimulate microglial activation, while GCs may inhibit it in balance.

## 10 Conclusion

At this stage, it is not possible to draw the conclusive remark regarding the interactions of these two systems for microglial conditions. With any settings applicable, we suggest that the HPA axis may play inhibitory, while LC-NA stimulatory, roles on microglia, respectively. In particular, the microglial conditions may be well-controlled only when the HPA axis and the LC-NA system orchestrate the regulation each other in terms of systemic homeostasis (Fig. 3). With the balance between HPA axis and LC-NA system well maintained, microglia may remain quiescent and play significant roles. However, under pathological conditions, this balance may be impaired, occasionally functioning in an opposite direction.

## Declaration of competing interest

The authors declare no conflicts of interest associated with this manuscript.
